# Supplementary material for: Knowledge of Tuberculosis preventive treatment among people living with HIV: A cross-sectional survey in selected regions of Tanzania
Source: PLoS One. 2024 Aug 1;19(8):e0307670. doi: 10.1371/journal.pone.0307670 (PMC11293724; doi:10.1371/journal.pone.0307670)
Supplement: Attachment — Submitted filename: Response to Reviewers.docx [file pone.0307670.s001.docx]

Dear PLOS team,

Thank you for working on my submission. I am humbled to provide feedback on the issues highlighted by the editors and reviewers. The same areas with issues are highlighted in the document with track changes.

| **Issue** | **Authors feedback** |
| --- | --- |
| **Additional Editor Comments** |  |
| **Major comments** |  |
| The calculated sample size for this study is 427 and the number of participants enrolled was 391. Not meeting the sample size means the results lack statistical power. | We appreciate the insightful comment regarding our study sample size. We acknowledge the discrepancy between the calculated sample size of 427 and the enrollment of 391 participants, which falls slightly short of the intended target. Despite our efforts, logistical challenges during data collection hindered us from reaching the desired sample size within the allocated timeframe. While we recognize the importance of statistical power, we employed rigorous analytical methods to mitigate the impact of the sample size limitation on the validity of our findings. Although the achieved sample size may have slightly reduced statistical power, we believe our results still provide valuable insights on the research questions addressed. |
| Another important factor to consider when evaluating knowledge about TPT is, "Time since enrolling into care" It is likely that those who have been in care longer and those who have actually received the TPT will be more knowledgeable. This variable needs to be evaluated. | We are thankful to the editor for this observation. The “Duration in care’’ variable was included and analyzed in this manuscript. This variable appears in the last column in Table 1, 3 and 4. We believe the misunderstanding may have been caused by improper wording of this variable. We have revised it to make it read “Duration in HIV care”. The same variable appears in the discussion section in lines 247-248. |
| There needs to be stronger justification by the authors why they think that PLWHIV should be knowledgeable about TPT. Patients do not prescribe this modality to themselves so knowledge about TPT is the purview of healthcare workers and not individuals that have received a diagnosis of HIV. | Given the heightened risk of progression from latent to active TB disease among People Living with HIV, possessing comprehensive knowledge about TB preventive treatment is important within this demographic. Such knowledge plays a role in mitigating misinformation, enhancing adherence, fostering trust, and reinforcing uptake of TB preventive services. Moreover, understanding the treatment course correlates positively with improved health outcomes. In that regard, our study aimed to evaluate the level of knowledge regarding TB preventive treatment among PLHIV populations in Tanzania, recognizing its significance in fostering positive health outcomes within this vulnerable group. |
| **Minor comments** |  |
| Consider having the paper reviewed for grammar. There are some grammatical errors that need to be corrected e.g. line 67, "Daily" should be, "daily" line 87, "Prevention" should be, "prevention" | We are thankful for the editor for the observation, we regret for not making a thorough grammatical check of this work. We have editor the entire manuscript for grammar. |
| Line 113: Define the abbreviation CTC in the paper before using it. | Thanks to the editor, the term has been defined as Care and Treatment Clinic (CTC). Additionally, we have navigated through the entire manuscript to make sure no other abbreviation is used without a full description |
| IPT and TPT cannot be used interchangeably. This paper evaluated IPT knowledge. IPT is a strategy for TPT. So, the title is not precise because it refers to TPT while the study evaluated IPT. Therefore, even in the paper, the use of the acronym TPT has to be precise: line 117 and page 15 of the paper. | We extend our gratitude to the editor for highlighting this critical issue. However, we regret the confusion caused by interchanging the concepts of TPT and IPT within the manuscript. As a result, we have undertaken a thorough revision of the entire manuscript to ensure alignment of the terms with their accurate definitions. Furthermore, we have opted to utilize TPT as the overarching concept in the title, while specifying IPT as a subset within the methods, results, and discussion sections, as our paper primarily focuses on evaluating IPT knowledge. The necessary amendments have been implemented in the specified sections, including lines 68, 92-93, 243, 244, 250, 251, 256, 257, 287, and 292. |
| In line 142 please state who (9) and (10) are then reference them. | The names Kazaura and Olum have been added in line 142 as advised by the editor. |
| **REVIEWER 1** |  |
| The manuscript lacks context about the TB and HIV situation in Tanzania.  In lines 65-66 it is written "In 2022, the proportion of people with TB coinfected with HIV stood at 17%". Regarding this study, much more important information would be what proportion of HIV-infected persons have (had) active TB and what proportion of HIV-infected persons have latent TB infection? | We are grateful for this critical observation, we have added in the Introduction part, the HIV, TB situation in the country and HIV/TB coinfection according to available literature. The amendments can be found in lines 65-71 |
| The authors do not mention anywhere the prevalence of latent TB infection and its diagnosis in Tanzania. Preventive treatment is given in the case of latent TB infection. Clarity is needed. | We have added this information in our manuscript. Thanks to the reviewer. The details about Latent TB in Tanzania is found in lines 71-73 |
| In lines 66-69, the statement "The National TB and Leprosy Program (NTLP) adopted the WHO recommendations for TB prevention among high-risk populations thus recommending daily Isoniazid as the main preventive drug for PLHIV and under-five household contacts of people with active TB "unclear. Different situations are discussed in one sentence - HIV infection (with latent TB infection) and contact persons with a patient with active TB. Need to clarify. | We regret improper wording in the highlighted sentences which lead to this confusion. We have revised the sentences in this paragraph to make them clear as advised by the reviewer. |
| **Minor concerns** |  |
| In line 70, the abbreviation ISP is not expanded. | We are thankful for this observation; this abbreviation has been removed. |
| In line 14 is probably misspelled "Tanzani". | Thanks to the reviewer, we have revised this to read as Tanzania |
| **REVIEWER 2** |  |
| This study examines knowledge of TB preventive treatment among a sample of people living with HIV in Tanzania. Overall, the study was very well written and easy to understand. The study findings were presented in a clear way and all tables were well structured and easy to interpret. The study discusses an important topic in HIV prevention, and the authors give plausible explanations of the key determinants of TPT knowledge that their study identified (e.g., gender, location etc). I have no additional comments. The paper was well written and it successfully addressed its central questions. | We are grateful for this insight. |

We are pleased to present our sincere gratitude for the efforts taken to locate issues in our manuscript. We appreciate your time and dedication to this work and look forward to hearing from you soon.

Warm regards,

Felix
