# Supplementary material for: Knowledge of Tuberculosis preventive treatment among people living with HIV: A cross-sectional survey in selected regions of Tanzania
Source: PLoS One. 2024 Aug 1;19(8):e0307670. doi: 10.1371/journal.pone.0307670 (PMC11293724; doi:10.1371/journal.pone.0307670)
Supplement: Attachment — Submitted filename: Response to Reviewers.docx [file pone.0307670.s002.docx]

Dear PLOS team,

Thank you for working on my submission. I am humbled to provide feedback on the issues highlighted by the editors and reviewers. The same areas with issues are highlighted in the document with track changes.

| **Issue** | **Authors feedback** |
| --- | --- |
| **Editor Comment** |  |
| Authors need to include the limitation in the manuscript that there is a reduction in the power of the results which was a result of not meeting the sample size. | We are thankful for this comment, the limitations section in the manuscript has been revised to show the reason to why the sample size was not attained in this study. |

We appreciate your time and support towards a successful publication of this work and look forward to hearing from you soon.

Warm regards,

Felix
